# Locomotion Disorders and Skin and Claw Lesions in Gestating Sows Housed in Dynamic versus Static Groups

**DOI:** 10.1371/journal.pone.0163625

**Published:** 2016-09-28

**Authors:** Emilie-Julie Bos, Dominiek Maes, Miriam M. J. van Riet, Sam Millet, Bart Ampe, Geert P. J. Janssens, Frank A. M. Tuyttens

**Affiliations:** 1 Institute for Agricultural and Fisheries Research (ILVO), Animal Sciences Unit, Scheldeweg 68. 9090 Melle, Belgium; 2 Ghent University, Faculty of Veterinary Medicine, Department of Reproduction, Obstetrics and herd health, Salisburylaan 133, 9820 Merelbeke, Belgium; 3 Ghent University, Faculty of Veterinary Medicine, Department of Nutrition, Genetics and Ethology, Heidestraat 19, 9820 Merelbeke, Belgium; University of British Columbia, CANADA

## Abstract

Lameness and lesions to the skin and claws of sows in group housing are commonly occurring indicators of reduced welfare. Typically, these problems are more common in group housing than in individual housing systems. Group management type (dynamic versus static) and stage of gestation influence the behavior of the animals, which in turn influences the occurrence of these problems. The present study compared prevalence, incidence and mean scores of lameness and skin and claw lesions in static versus dynamic group housed sows at different stages of gestation during three consecutive reproductive cycles. A total of 10 Belgian sow herds were monitored; 5 in which dynamic groups and 5 in which static groups were utilized. All sows were visually assessed for lameness and skin lesions three times per cycle and the claws of the hind limbs were assessed once per cycle. Lameness and claw lesions were assessed using visual analogue scales. Static groups, in comparison with dynamic groups, demonstrated lower lameness scores (P<0.05) and decreased skin lesion prevalence (24.9 vs. 47.3%, P<0.05) at the end of gestation. There was no difference between treatment group regarding claw lesion prevalence with 75.5% of sows demonstrating claw lesions regardless of group management. Prevalences of lameness (22.4 vs. 8.9%, P<0.05) and skin lesions (46.6 vs. 4.4%, P<0.05) were highest during the group-housed phase compared to the individually housed phases. Although the prevalence of lameness and skin lesions did not differ three days after grouping versus at the end of the group-housing phase, their incidence peaked during the first three days after moving from the insemination stalls to the group. In conclusion, the first three days after grouping was the most risky period for lameness incidence, but there was no significant difference between static or dynamic group management.

## Introduction

Since January 2013, all sows in the European Union must be group-housed from four weeks after service to one week before parturition (European Directive 2001/88/EC). Group housing allows for social contact and interactions between sows [1;2]. Moreover increased activity of group housed sows as compared to individually housed sows, has a positive effect on muscle and bone development [3;4]. However, the positive benefits to group housing of sows may also be accompanied by factors that negatively impact sow welfare. Depending on the feeding system, more feeding competition may occur, and aggression towards other sows increases, resulting in an increased risk for skin lesions, vulva biting, claw problems and lameness [1;5;6]. In commercial settings, sows will not only be housed in groups, but will also experience different types of housing through gestation including insemination and farrowing crates. Gestating sows are often housed in larger groups than feral pigs, with (somewhat to completely) unfamiliar animals and these group compositions are usually changed at least once per reproductive cycle [7]. When housing sows in groups, many aspects need to be considered, including group size, group density, pen design, floor type, bedding material, feeding system and group management (dynamic versus static) [8]. Commercial sow groups can be managed as either a static or a dynamic group. In static groups the group composition stays the same after formation, so only one breeding group is present per pen. sows experience one bout of mixing, and the associated aggression, at the beginning of gestation. If a sow recycles or is removed for some reason, no replacement sow is introduced. In dynamic groups, animals are introduced into and removed from the group throughout the gestating period, with the number of introductions and removals dependent upon individual farm Lameness is prevalent in group housed sows, as shown by Pluym et al. [9] who found a lameness prevalence of 9.7% in Belgian herds. Comparable findings for lameness prevalence are found in Finland (8.8%), Norway (13.1%) and the United Kingdom (14.4% in gestating gilts and 16.9% in gestating sows) [10–12]. Dynamic groups have more than one breeding group housed in a pen together at the same time. This approach uses pen space more efficiently compared to static groups. However, sows are exposed to multiple bouts of mixing and the associated aggression throughout gestation every time a new group of sows is introduced. Aggression is unavoidable when group housing pigs, because they will inevitably fight in order to establish a dominance hierarchy [13;14]. Skin lesions are often a result of aggression [15]. Due to the more frequent mixing bouts in dynamic sow groups, more aggressive behavior and therefore more skin lesions might be expected [2;16–19]. As soon as the social hierarchy is established, the fighting decreases and aggressive behavior can be kept to a minimum in well managed and well-designed housing systems [8]. The unrest and agonistic behavior associated with such changes result in more skin lesions and locomotion disorders, including claw lesions [15;17].

Locomotor disorders (lameness) are the second largest reason for early culling of sows [9]. Since the mandate for group housing of gestating sows in the EU the prevalence of these disorders has increased [20;21]. Lameness can be described as the clinical appearance of locomotion disorders that might result in pain, discomfort and impaired mobility, depending on the severity and type of disorder [22]. Lameness may also reduce general activity, social behavior and exploration, as reviewed by Weary et al. [23]. Many factors may influence the development of locomotor disorders in breeding sows. Lameness can have several non-infectious risk factors, such as osteochondrosis and limb malformation, and infectious risk factors such as joint arthritis or infected skin lesions [10;20;24–26]. Various studies have reported that management, breeding age, parity, claw lesions, feed, floor properties of the pen and rearing strategies are important risk factors [11;23;27;28]. Furthermore, sows housed in groups have a higher risk of lameness resulting from fighting due the aggression related to competition for feed or (re)grouping [29–31]. Lameness has an economic impact, as it decreases reproduction performance and longevity and increases human workload and veterinary costs [32–34].

Locomotion disorders can be associated with claw health, such as injuries to the sole, wall, white line and heel [24;35]. Claw lesions, their causes and consequences have been studied extensively and the shift to group housing has resulted in more attention for this problem [5;36–40]. Claw lesion prevalences of 60 to 95% have been reported [9;40]. Claw lesion etiologies are complex and multifactorial but some studies suggest that genetics, housing, nutrition and facility management all play a role [41–43].

Long-term sow observation and evaluation are needed to understand the impact of group management and associated housing methods on the evolution of leg and claw problems at different phases within the reproductive cycle. Prevalence measures give an overview of the occurrence of the actual problem and incidence measures (the number of newly diagnosed cases of a disease during a given period of time) allow for recognition of hazardous phases within the reproductive cycle of the sow. Mean locomotion scoring allows more accurate representation of the severity of the condition. Longitudinal studies allow to show the patterns of a variable over time, and to calculate incidences. The longitudinal, repeated measurements essential when incidences are being calculated. Although studies conducted by Pluym et al. [9] have investigated the short term effects of group management on the prevalence of lameness and claw lesions in Belgian pig herds, to date, there are no longitudinal studies evaluating sow group management on lesion and lameness incidence.

The aims of the present study were to compare the prevalence, incidence and mean scores of lameness and skin and claw lesions in static versus dynamic group housed sows at different stages of gestation during three consecutive reproductive cycles.

## Materials and Methods

The study was approved by the ILVO Ethics Committee (Reference 2011/153). Participating farms were fully aware of the study’s aims and objectives and gave permission to collect and publish the data conditional upon our promise not to reveal their identity. All identifying information regarding the participating farms therefore remains unpublished.

### Study population

**Fig 1:**
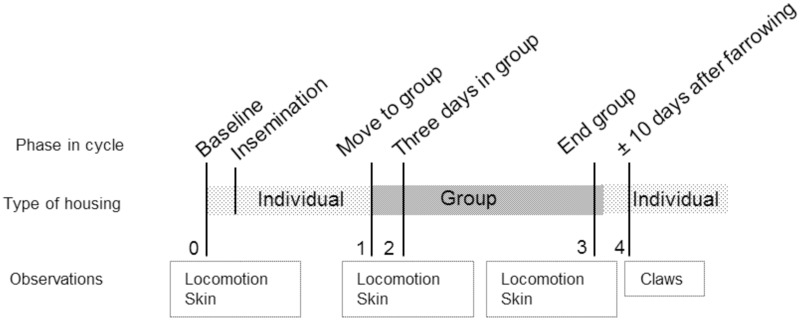
Timing of the various observations during each reproductive cycle of the sows. In addition to the first baseline measurement (0), locomotion and skin lesions were scored three times (1, 2, and 3) and claw lesions once (4) per cycle. Sampling point 1: prior to moving to the group. Sampling point 2: three days after moving to group. Sampling point 3: end of group housing phase, around d 108 of gestation. Sampling point 4: approximately 10 days after farrowing.

The present study was carried out on 10 commercial pig farms in Flanders, Belgium. Farms were selected based on their willingness to participate in this observational study. Farms were required to have a sow group housing system established for a minimum of 1 year, were not allowed to change group housing system during the study and were within 100 km from the ILVO institute, and there had to be an equal number of farms with dynamic and static group management. Farms’ individual characteristics are described in Table 1. The presence or absence of locomotion problems and skin or claw lesions was not taken into account when selecting the farms. Sows to be included in the study were randomly selected by age (using the randomization function in Excel) with a mean group size of 27 (group size range: 24–30) for a total of 138 and 132 sows in dynamic and static systems, respectively. At the onset of the study, the parity range was 1–6 (Table 1). Multiparous sows and gilts were combined and referred to as ‘sows’ for the remainder of the manuscript. Every sow of each of these groups was evaluated four times per cycle: locomotion and skin lesions were scored three times (sampling points 1, 2 and 3), whereas claw lesions were scored once per cycle (sampling point 4) (Fig 1). As farms differed in time schedule according to moving animals, time spent in specific areas of the farms differed, see Table 1 for number of days spent in the insemination stalls per farm.

**Table 1:** General characteristics of the 10 studied herds.

	Farm									
	1	2	3	4	5	6	7	8	9	10
**Group pen characteristics**										
**Group management**^a^	S*	S	S	S	S	D	D	D	D*	D*
**Group size (number of sows)**	70	80	170	48	56	80	46	20	58	72
**Feeding system in group pen**	VM	VM	VM	ESF	FAS	ESF	ESF	ESF	ESF	ESF
**m^2^/sow in group pen**	1.43	2.33	2.10	2.10	3.31	2.37	2.00	1.95	2.63	2.61
**Breed**	Seghers hybrid	Topigs	PIC	Crossbred York	Danbred	Topigs	Topigs	Crossbred York	Topigs	PIC
**Number of days in insemination stalls**^b^	30	28	28	25	35	3	28	5	4	28
**Batch production system (wk)**^c^	11	5	2	3	4	1	3	3	4	4
**Number of studied sows at baseline per parity**^d^										
**Parity 1**	3	14	12	9	9	25	8	5	18	12
**Parity 2–4**	27	11	15	16	16	0	21	13	12	20
**Parity ≥4**	0	0	0	0	0	0	0	9	0	0

VM = Vario-Mix feeder, ESF = electronic sow feeder; FAS = free access stalls

* Farm 1, 9 and 10 used straw bedding in the gestation unit

^a^ S = static groups; D = dynamic groups

^b^ Farm used different number of days that sows spent in the insemination stalls

^c^ Week batch production system for sows

^d^ Parity range at onset of the study was 1–6.

When sows were removed from the groups, no replacement sows were included in the study. Farmers remained in charge of making decisions about sow removals throughout the study.

All observations were performed by two experienced assessors who had been trained to use the scoring systems. Training involved repeated scoring of locomotion, skin lesions and claw lesions of sows by all assessors until inter-observer repeatability exceeded 90%.

### Locomotion

**Fig 2:**
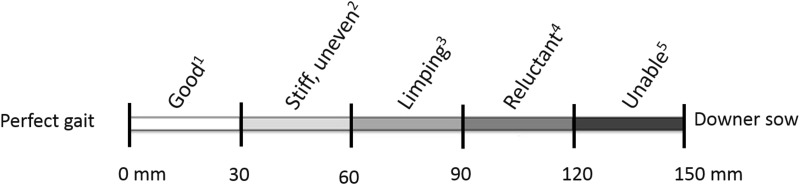
Lameness classes on the tVAS. Explanation of scores: 1. ‘Good’: even stride, ease of movement. Little inducement needed, comfortable on all feet. 2. ‘Stiff, uneven’: movement is not fluid, uneven strides, stiffness. 3. ‘Limping’: lame in one leg, limping. Shortened stride. Compensatory behaviors (dipping of head, caudal swagger, arched back). 4. ‘Reluctant’: reluctant to place weight on affected limb(s). Reluctant to walk. Lame in more than one leg. Caudal swagger. 5. ‘Unable’: does not place affected limb on floor. Very unwilling to move, does not walk. A vertical mark along the tVAS can be placed to score a sow. tVAS = tagged visual analogue scale.

A baseline locomotion score was recorded before the first service within the study (day 0) and further locomotion scoring took place at sampling points 1, 2 and 3 (Fig 1). Locomotion was scored in the corridor behind the insemination crates or, during the group housed period of the reproduction cycle, locomotion was scored while a sow was walking in her home pen. If the sows needed to be encouraged to move, a person walked beside them and gave either vocal cues or waved his/her hands. Locomotion was scored using the 150 mm Visual Analogue Scale with labels (tVAS) developed by Nalon et al. [44] (Fig 2). For the condition in question, the observers put a vertical mark across the tVAS in the position corresponding to their perception of the sows’ gait. Locomotion of sows was analyzed as mean locomotion score, lameness prevalence and lameness incidence. For estimating lameness prevalence, sows were considered lame when their locomotion score was >60 mm on the tVAS [45]. For estimating lameness incidence, a new case of lameness was defined as a previously non-lame sow (<60mm on tVAS) that had become lame (>60mm) and for which the locomotion score had increased by >30 mm since the previous score. Incidence was calculated per day at risk.

### Skin lesions

Skin lesions were scored at the same time as locomotion scoring (Fig 1) by using a slightly adjusted scoring method from the Welfare Quality^®^ protocol for pigs [46]. The left side of the sows’ body was visually divided into five regions: ears, front (head to back of shoulder), middle (back of shoulders to hindquarters), hindquarters and legs (from accessory digit and above). A 3-point ordinal scale was used to score skin lesions on each body region, with a score A indicating 0 to 4 visible skin lesions, score B indicating 5 to 10 lesions and score C indicating >10 lesions. These scores were summarized by assigning a binary total body score. If score A was recorded on all body regions, a total score of 0 was assigned. If at least one score B or C was recorded, a total score of 1 was assigned (e.g. positive score for the presence of skin lesions). Skin lesions of sows were analyzed in terms of the prevalence and incidence of skin lesions.

### Claw lesions

**Fig 3:**
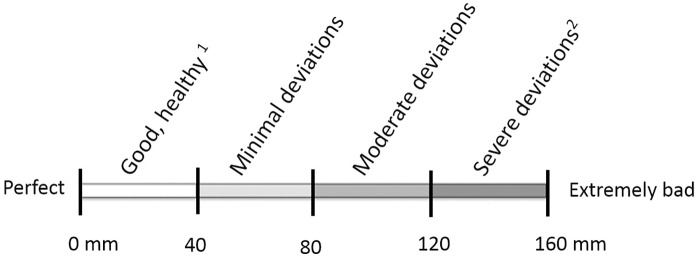
Claw lesions classes on the tVAS. Eight claw parameters were used: 1) heel horn, 2) heel/sole crack, 3) white line, 4) skin lesions between the coronary band and the origin of the dewclaw, 4) horizontal cracks in the wall horn, 5) vertical cracks in the wall horn, 6) claw length and 7) dewclaw length. Explanation of scores depends on the claw parameter, but all scores vary between 0 mm (1) (a perfectly, healthy claw without any deviations, erosion, cracks or deviations in length and 160 mm (2) (being a claw in terrible state, including for example severe erosion, cracks, inflammations and loo long or (partly) ripped-off (dew)claws). A vertical mark along the tVAS can be placed to score a claw parameter. tVAS = tagged visual analogue scale.

Hind claws of the sows were visually assessed around 10 days after parturition (sampling point 4; Fig 1) while the sows were housed in the farrowing crates. During the time in the farrowing crates sows are individually housed, cannot walk away and lay down more calmly while suckling their piglets, which enables close inspection of the claws from all sides. When the sows were standing, not all parameters were immediately scored but as soon as the sows lay down, the remaining parameters were scored. Claws were scored using a recording system based on the “*Zeugenklauwen Check*” by Wageningen University Livestock Research [47] and the ‘FeetFirst’ method by ZinPro [48], as described in Bos et al. [30]. Eight claw parameters: 1) heel horn, 2) heel/sole crack, 3) white line, 4) skin lesions between the coronary band and the origin of the dewclaw, 5) horizontal cracks in the wall horn, 6) vertical cracks in the wall horn, 7) claw length and 8) dewclaw length were evaluated using a visual guide to the type and severity of the lesions and erosion. Instead of the ordinal scale presented in literature, we used a 160 mm t VAS (Fig 3). The length of the dewclaw was determined by pushing the dewclaw against the claw to be able to compare dewclaw length and heel height. For each claw parameter a mean score per sow/parameter/inspection was calculated. Results of claw lesion scores are presented as a mean score per sow per parameter and as claw lesion prevalence. A sow was defined as having a claw problem if the tVAS score was >80 mm for at least one parameter per sow.

### Statistical analysis

The mean locomotion and claw lesion scores were analyzed using a linear mixed regression model with group management type (static or dynamic groups), phase of the reproductive cycle, their interactions and parity as fixed effects and farm and sow as random effects to correct for the repeated measurements. Non-significant interactions were excluded from the final models. The dichotomized skin lesion score and prevalence of locomotion and claw problems were analyzed using similar logistic mixed regression models with the logit link. The analyzed continuous data were considered to be sufficiently normally distributed, based on the graphical evaluation (histogram and QQ-plot) of the residuals. In case of post hoc pairwise testing, p-values were corrected with the Tukey-Kramer adjustment for multiple comparisons. Incidence of lameness and skin lesions was calculated per day at risk. For the analysis of the incidence of lameness and skin lesions, only numerical results are provided. All analyses were performed using *proc* GLIMMIX in SAS 9.4 (SAS Institute Inc., Cary, NC, USA). Results are given as LS-mean ± SE.

## Results

### Study population

For the total duration of the study 55.2% of the sows of the initial experimental groups were removed; 42% of removed animals came from static groups and 58% from dynamic groups. Reasons for removal were specified by the farmers as locomotor disorders (9%), reproductive failures (56%) and other or unknown reasons (35%).

### Mean locomotion score

**Fig 4:**
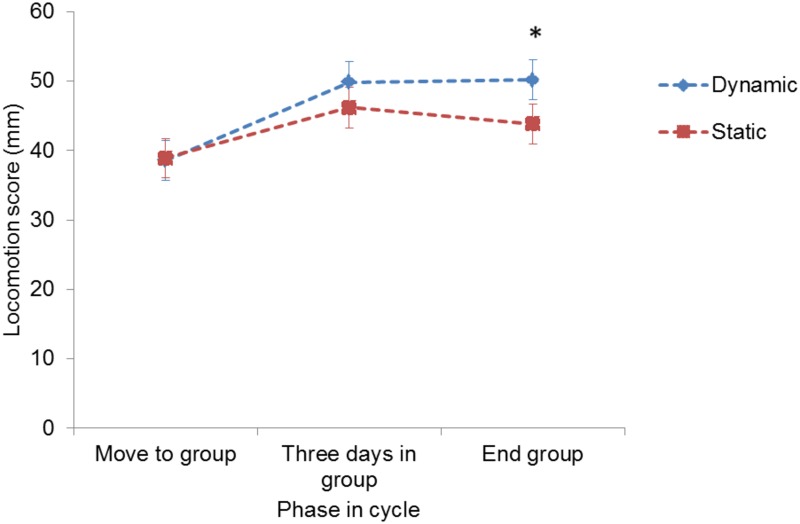
Effect group management of sows on mean locomotion score at different stages in the reproductive cycle. Results are given as LS-Mean ± SE. * indicates significant differences between group management per phase in the cycle (P<0.05).

The interaction between phase in the cycle and group management had an effect (P<0.001) on mean locomotion score with greater locomotion scores at the end of the group housing period in dynamic vs. static groups (Fig 4). There was a tendency for a parity effect on locomotion score (P = 0.068) with a decrease of 1.85 mm per parity level.

### Lameness prevalence

**Fig 5:**
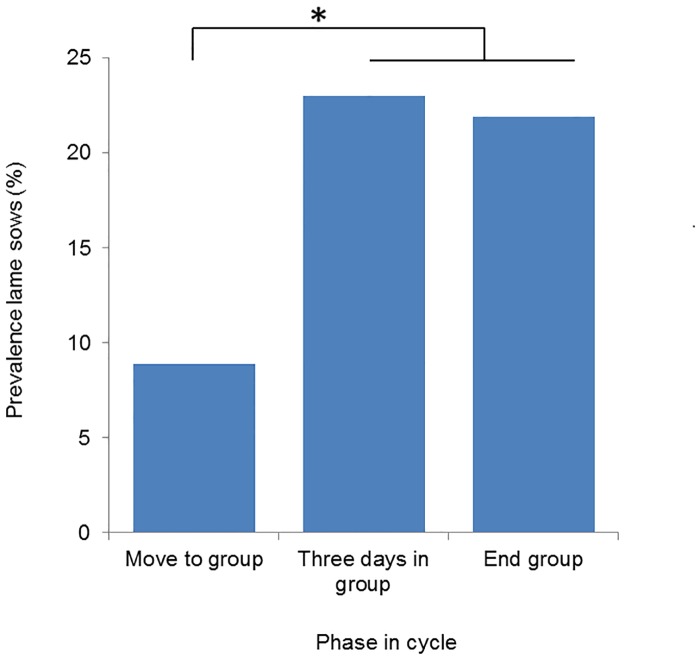
Percentage of lame sows per phase in the reproductive cycle. * indicates significant differences between phases in the cycle (P<0.05).

The interaction between phase in the cycle and group management had no effect on lameness prevalence (P = 0.477), nor was there a parity effect (P = 0.527). Phase in cycle had an effect on lameness prevalence. There was a lower prevalence when sows were moved to the group pen (8.9%) compared to three days after grouping (23.0%, P = 0.040) and compared to the end of the group housing period (21.9%, P = 0.006) (Fig 5).

### Lameness incidence

**Fig 6:**
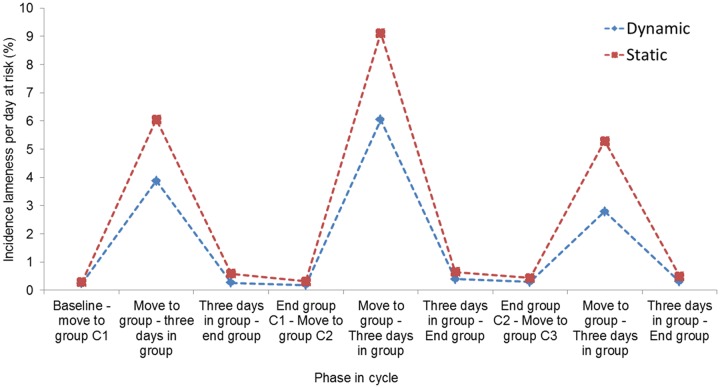
Incidence of lameness per day at risk in sows in static versus dynamic group management systems at different stages in the reproductive cycle.

Lameness incidence was the highest between move to group and three days after grouping regardless of management systems (Fig 6). The evolution of lameness incidence throughout the reproductive cycle is largely similar for both management systems.

### Prevalence of skin lesions

**Fig 7:**
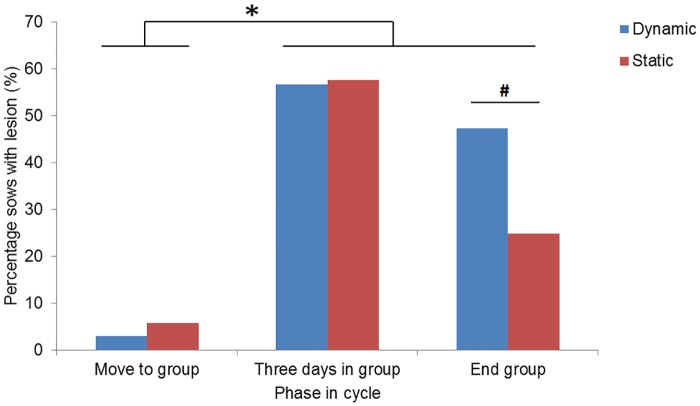
Prevalence of sows with skin lesions per phase in the reproductive cycle and for dynamic versus static group management separately. * indicates significant differences between phase in the cycle (P<0.05). ^#^ indicates trend towards differences between group management systems (0.05<P<0.01)

The interaction between phase in the cycle and group management had an effect on skin lesion prevalence (P<0.001). Skin lesion prevalence tended to be higher in dynamic groups (47.3%) as compared to static groups (24.9%, P = 0.061) at the end of the group housing period, but did not differ during other phases of the reproductive cycle (Fig 7). Irrespective of type of group management, very few sows had skin lesions at move to group (Fig 7). Three days after grouping, however, more than half the sows had skin lesions. Parity significantly affected skin lesion prevalence (P<0.001); the lower the parity the higher the prevalence.

### Incidence of skin lesions

**Fig 8:**
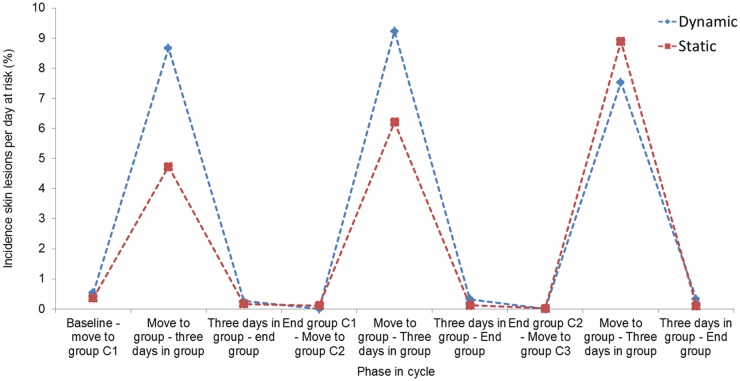
Incidence of skin lesions per day-at-risk for dynamic versus static group management.

Incidence of skin lesions was the highest from grouping to three days after grouping for both group management systems (Fig 8).

### Mean claw lesion score

**Fig 9:**
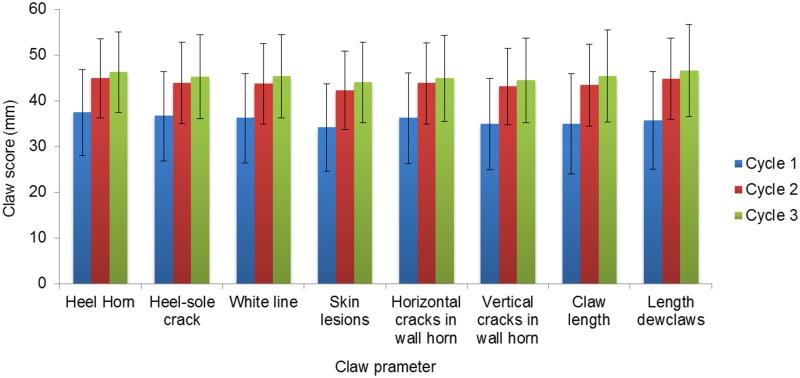
Mean claw scores in sows per claw parameter per cycle. Results are given as LS-Means ± SE.

There were no differences in mean scores per claw parameter between the two group management systems (P>0.1 for all 8 claw parameters). Mean scores per claw parameter per monitored cycle are shown in Fig 9. Parity affected the heel horn (increase of 2.89 mm on tVAS per increasing parity, P<0.001) and claw length score (increase of 3.22 mm on tVAS per increasing parity, P = 0.009). There was a tendency for a parity effect for skin lesions around the claw (increase of 1.75 mm on tVAS per increasing parity, P = 0.054) and length of the dewclaw (increase of 2.50 mm on tVAS per increasing parity, P = 0.073). A significant effect of the monitored reproductive cycle for all claw parameters (P<0.050) was observed, except for the heel sole crack; the higher the monitored cycle, the higher the mean claw score.

### Prevalence of claw lesions

The overall prevalence of claw lesions was 75.5%. No effect of group management (P = 0.613) or monitored cycle (P = 0.303) was observed. The prevalence of claw lesions increased with increasing parity (P = 0.004).

## Discussion

The present study showed that at the end of the group housing phase the mean locomotion score, lameness incidence and skin lesion prevalence were lower when sows were housed in static versus dynamic groups. We found no effect of group management on claw lesions nor on both lameness and skin lesion prevalence. According to the incidences, the first few days after grouping have a pronounced detrimental effect on the development of locomotion problems and skin lesions for both static and dynamic groups of sows.

### Locomotion

The mean locomotion score tended to be better for sows with a higher parity. This is consistent with results from other research [10;41;49]. However, the decreased risk for a higher locomotion score by aging can be the results of the culling strategy, because all unhealthy or weak sows are culled, leaving only the more robust and healthy sows in the herd. This is an problem inherent to studies performed under commercial circumstances. During the present study, 55.2% of the sows were removed or euthanized over three reproductive cycles, typical for a commercial situation [33]. Under commercial circumstances sows are often culled after farrowing or weaning but this depends on the reason of removal. This could possibly have influenced our study as well [50;51].

The present study showed that around 22% of the sows were lame (at least 60 mm on the tVAS) while housed in the group pens. This means that they are lame in at least one leg, obviously limping and showing compensatory behavior. No difference between the two group management systems was found, and lameness prevalence varied widely between herds subjected to the same group management type. Lameness prevalence in the current study was higher than reported than prevalences reported for Finland (8.8% lame sows) and Norway, where 13.1% of the loose-housed dry sows showed lameness in a hind leg [10;12]. The results of Pluym et al. [9] confirm wide variation between herds in terms of lameness prevalence.

The peak in lameness incidence was found from immediately prior to move to group until three days after moving, irrespective of group management, this in agreement with the results of Bos et al. [30]. Numerically higher incidences were found in dynamic groups. Li and Gonyou [49], reported increased cases of lameness in a dynamic group compared to a static group after the group housing period. This can be explained by the increased number of introductions of new sows into dynamic groups, which induces aggression and increases risk of injuries due to fighting [52;53]. In both types of group management systems, but in dynamic groups in particular, the first days after grouping are by far the most risky period for sows to develop lameness. This is to be expected, as aggression between sows is greatest when sows are first introduced to each other and they fight to form hierarchies, often resulting in locomotor problems [54]. The incidence during the other phases in the reproductive cycle was much lower, also in dynamic groups. This is unexpected, as we hypothesized that in dynamic groups regular interactions between sows would occur throughout the entire group-housed period.

Interestingly, both the prevalence and mean score of lameness did not differ between the start and end of the group housing phase, while the incidence differed greatly. This shows the added value of using all three methods of measuring locomotive disorders. Prevalence of lameness was very low at the end of the time spent in the insemination crates. A reason for this can be that when sows are housed individually, no interactions can take place with other sows, and the sows do not have to cover distances in the pen in order to eat, sleep or defecate and urinate [54;55]. Diminished locomotion and interaction both decrease the risk of becoming lame or maintaining locomotor problems. In our study it appeared that sows recovered spontaneously from lameness during the time spent in individual housing. Research has shown that pigs with clinical signs of lameness can recover spontaneously when housed in pens where they can eat and drink without the need to compete with healthy sows, e.g. individual housing [56;57].

### Skin lesions

The first few days after moving to the group and the entire period of group housing appear to be critical for sow welfare given the high prevalence and incidence of skin lesions found in this study. The trend towards more lesions for sows in dynamic groups at the end of the group housing period is in agreement with our hypothesis. Total skin lesion scores were higher three days after move to group and at the end of the group phase compared to the moment before grouping; this is in agreement with other research [13;17;58]. Newly-grouped animals engage in aggressive behavior to form a social hierarchy, which is reflected in the number of skin lesions [15;59]. Our study confirmed our hypothesis regarding aggressive behavior; at the end of the group-housed phase the prevalence of skin lesions was lower for static groups.

Numerically higher skin lesion incidences were found in dynamic groups in the first and second monitored reproductive cycle during the first three days of group housing compared to static groups. This is in accordance with the research of Li and Gonyou [49], who reported an increased number of skin injuries in sows observed before farrowing in a dynamic group compared to a static group. Strawford et al. [60] found no differences in aggression, skin lesions and cortisol concentrations between sows in static and dynamic groups. In the third monitored cycle of the present study the incidence during the first three days of group housing was higher for static groups, which differs from the first and second cycle. Selective culling of sows might be a possible explanation for this shift in incidence, besides in the third monitored cycle the sample size was smaller compared to the first and second cycle.

Social interactions between pigs can originate from heritable traits; in our research we assessed sows with a different genetic background. Genetic selection generally highlights physical traits of production, like growth and litter size, but the transition to group housing adds the requirement that sows can live peacefully in groups. Social behavior should therefore be considered as an important and highly significant trait as well [61].

The time spent separated in the gestation stalls after artificial insemination varied among the 10 farms in this study. This may have influenced our results, as there is evidence that the stage of reproductive cycle at the moment of entering the group may affect aggression [49;54]. Besides, to maintain stable relationships (i.e., maintaining a dominance hierarchy), sows must have the ability to recognize individual members of the group. Research shows that large group sizes hinder easy recognition and could disturb the development of hierarchies [62;63]. In addition to group size, the duration of having been separated is an important factor for sows to be able to recognize conspecifics although it is known that sows are able to recognize each other for several weeks after separation [59;64].

The farms in our study used sows of different parities within a group, which also affects the aggressive behavior in a group. If no older sows are present, sorting by parity might be a valuable method to protect the most vulnerable young and small sows from severe injuries caused by aggression at grouping [65]. Literature suggests that in mixed parity groups, pen design can be improved, e.g., by increasing the amount of space per sow and providing places or barriers where vulnerable or weaker animals can go to get away from other (aggressive) animals [17;65]. Group housed sows would therefore benefit from more research into the effect of parity division within the group, stage of reproductive cycle when grouping and genetics as a contributing factor to aggressive trait characteristics.

### Claw lesions

The prevalence of claw lesions (75.5%) corresponds to the reported prevalence of 60–90% in various other sow studies [5;12;34]. The causes of claw lesions are multifactorial, including genetics, nutrition, age, parity, earlier experience, and management and housing [5;42;66]. We found that the prevalence of claw lesions increases with increasing parity. Several other studies confirm that the prevalence increases with the age of sows [5;9]. A temporary decrease in prevalence during the lactation period has been reported as well [9]; we were unable to test this because we assessed the claws only once per cycle in this study.

### Strengths and weaknesses of the study

Prevalence was calculated for all three output variables (locomotion, skin and claw lesions); for locomotion and skin lesions, incidence was also calculated. Prevalence and incidence are the basic measures of disease frequency [67]. The prevalence is the total number of cases in the population at a specific moment in time. The incidence is the number of new cases in the population per day at risk. Incidence is thus a valuable measure to evaluate the risk of getting a specific disease, but it requires a longitudinal study and repeated monitoring. The present longitudinal study, in which we monitored specific parameters (locomotion and skin lesions) repeatedly over time in the sows, allowed us to study the process of change over time, and the effect of sow-specific factors (such as locomotion and skin lesions) as well as management-specific factors (such as group management). Because longitudinal data collection is time-consuming we had to limit the total number of farms that could be monitored.

To calculate prevalence and incidence of a condition, a cut-off point in the scoring systems is needed to assess whether an animal is affected with the condition or not. For lameness, no unequivocal cut-off between “lame” and “non-lame” can yet be determined, as some authors consider that some changes in the locomotion pattern (e.g., stiffness) might not result in discomfort or pain [66;68] and therefore should not be considered as lame. In the present study we used a cut-off based on the lameness score at which sows became less willing to walk to obtain a highly tempting reward [45]. Using the mean locomotion score allowed us to describe the severity of lameness in a more nuanced way than classifying sows simply as lame vs. non-lame. However, VAS scores might lead to overestimation of the clinical importance of small differences, e.g. the tendency of a parity effect on locomotion score with a decrease of 1.85 mm per higher parity. A statistical significance does not necessarily mean that there is a biological relevance as well [69]. This biological impact on the sows is more important, and should be taken into account when assessing these statistical outcomes.

Field studies like this one provide valuable information about incidence, prevalence and severity of lameness and claw and skin lesions in practice, but the high variation among farms might limit the power to detect significant differences. A certain variation between farms is needed in order to identify risk factors for specific disorders. However, the observed farms differed so much for many factors that too few replications for each of these factors were available in order to test all their effects, besides this was not the aim of our study. Both dynamic and static group management systems are associated and therefore co-varying with their own, particular characteristics. The effect of each of these variables cannot be determined independently of group management system. For example, in dynamic groups more electronic feeding systems are being used compared to in static groups. This implies that we have to focus on the comparison between group management system as is used in practice, and that we cannot figure out exactly which aspects are responsible for the observed differences. All previous studies on the effect of group management differ in a number of other management procedures like the provision of bedding, type of flooring and space allowance [1;9;60]. In our study there was no consistent, convincing evidence that sow welfare was adversely affected in dynamic groups in comparison to static groups, considering the high variation between herds.

Due to experimental restrictions (i.e., need to follow farm protocols) all observations were performed in the home pen of the sows. This may have influenced the locomotion, skin and claw scores. Removing sows for ethical and economic reasons during the study period was inevitable and common practice under commercial settings. This longitudinal monitoring over three successive reproductive cycles under commercial circumstances resulted in a descriptive overview of the circumstances in the Flemish sow husbandry.

## Conclusions

The first three days immediately after (re)grouping are a very risk full period for developing lameness or incurring skin lesions independent of group management system. Our results show better mean locomotion score and skin lesions prevalence in static versus dynamic group-housed sows, but we did not find a group management effect on claw lesions. All three output variables (locomotion, and skin and claw lesions) in group-housed sows would benefit from more research on time and method of grouping, effect of parity division within the group, and genetics as a contributing factor to aggressive trait characteristics. Future research should focus on optimizing the housing environment and management of group-housed sows to reduce the risk of developing lameness or being wounded.

## Supporting Information

S1 Table Data of locomotion and skin lesion scoring. (XLSM)

S2 Table Data of claw scoring. (XLS)
